# Establishment of an *in vitro* aerobic bacterial community as a model of the human lung microbiome

**DOI:** 10.1128/msystems.00491-26

**Published:** 2026-08-03

**Authors:** Maria Kulosa, Matheus Regis Belisário-Ferrari, Florian Semmler, Mathilde Büttner, Corinna Duck, Zeinab Namazi, Nico Jehmlich, Martin von Bergen, Tobias Bonitz, Leonard Kaysser

**Affiliations:** 1 Pharmaceutical Biology, Institute for Drug Discovery, Medical Faculty, University of Leipzig, Leipzig, Germany; 2 Department of Molecular Toxicology, Helmholtz-Centre for Environmental Research–UFZ GmbH, Leipzig, Germany; Georgia Institute of Technology, Atlanta, Georgia, USA

**Keywords:** microbiome, lung microbiome, microbiology, bacterial consortia, artificial microbiome, co-culture model

## Abstract

The human lung microbiome is increasingly recognized as a key player in drug metabolism, yet it remains largely understudied. To replicate this complex physiological environment in a controlled setting, we developed a simplified artificial lung microbiome model composed of four representative bacterial species: *Pseudomonas koreensis*, *Rothia aeria*, *Neisseria cinerea*, and *Streptococcus downei*. We successfully established a stable 10-day co-culture at 34°C using brain heart infusion medium, as validated by quantitative PCR, viability PCR, and conventional microbiological methodologies. Integrated bioinformatic analyses revealed a variety of potential microbe-microbe interactions, which were supported by metaproteomic analysis using mass spectrometry. Our model provides a foundation for in-depth studies, such as, for example, the effects of pulmonary drugs on the lung microbiome, and how, in turn, the microbiome may influence therapeutic outcomes.

**IMPORTANCE:**

Once thought to be sterile, the lung microbiome is now understood to host a dynamic microbiome capable of influencing respiratory health and disease. Understanding interactions among the microbes within this community is essential, as these relationships may drive disease progression or foster resilience in both acute and chronic inflammatory conditions. We developed a reproducible lung microbiome model comprising *Pseudomonas koreensis*, *Rothia aeria*, *Streptococcus downei*, and *Neisseria cinerea*. Simplified models enable controlled studies to dissect specific microbial interactions, laying the foundation for insights into lung microbial ecology. In the future, more complex models will enhance our understanding of microbial roles in disease outcomes, with our platform serving as a basis for testing therapeutic strategies.

## INTRODUCTION

The human microbiome encompasses all the microorganisms residing in the various niches of the body. It plays a critical role in both maintaining health and in the development of diseases (1). The composition of the microbiome differs between individuals, influenced by factors such as diet, age, medication, and infections (2, 3). As a microbial habitat, the lung offers unique ecological conditions. Due to the bidirectional airflow that connects the oropharynx with the lungs, the respiratory environment is notably dynamic and transient (4). This anatomical connection facilitates the migration of microorganisms, directly affecting the composition of the lung microbiome. Additionally, the temperature gradient in the respiratory tract, which ranges from ambient air to core body temperature, creates a diverse habitat that is conducive to microbial life (5). However, unlike the nutrient-rich gut, the lung is a low-nutrient environment, and even small changes in oxygen tension, pH, blood perfusion, and alveolar ventilation can significantly impact the composition of the microbiome (6). While such environmental constraints are difficult to fully replicate *in vitro*, they underscore the importance of tightly regulated microbial interactions that enable colonization of this niche. This is partially mediated by the secretion of specialized metabolites, e.g., for competitive nutrient exclusion, biofilm formation, and quorum sensing (7).

The lung microbiome in healthy individuals consists of diverse microorganisms, including bacteria, with the most prevalent phyla being *Pseudomonadota*, *Actinomycetota*, and *Bacillota* (8). This closely mirrors the situation in the upper respiratory tract and oral cavity, supporting the hypothesis that the lung microbiome is transient, with microorganisms primarily migrating from these adjacent regions (9). However, current perspectives propose that the “health” of a microbiome is better characterized by its functional metabolic capacity rather than simply by the presence of specific bacterial species (10). In a recent study, we investigated the metabolic potential of the lung microbiome, identifying the highest diversity of biosynthetic gene clusters (BGCs) within the phyla *Pseudomonadota*, *Actinomycetota*, and *Bacillota* (11). Using a sequence similarity network and reference gene clusters, we linked many of these BGCs to putatively beneficial roles in the lung environment, such as osmo-adaptation, surfactant production, and protection against oxidative stress.

The development of artificial microbiome models has improved our ability to investigate microbial community dynamics and their functions under controlled conditions. These models, especially for the gut and skin, provide critical insights into drug-microbiome interactions, microbial ecology, and their potential therapeutic applications (12–15). Building on these advancements, we report the development of the first reproducible artificial human lung microbiome model, capable of sustaining a stable co-culture for up to 10 days. By combining *in vitro* microbiological techniques with bioinformatic analysis and metaproteomics, we successfully elucidated potential microbe-microbe interactions within this model.

## RESULTS AND DISCUSSION

### Composition of the microbial consortium

To establish a tractable co-culture model of the human lung microbiome, we selected four bacterial strains according to three criteria: (i) documented relevance to the respiratory tract, (ii) complementary metabolic capabilities, and (iii) suitability for routine handling under biosafety level 1 (BSL-1) conditions. Guided by recent literature, including the work of Semmler et al. (11), we chose *Pseudomonas koreensis* DSMZ16610, *Rothia aeria* DSMZ14556, *Streptococcus downei* DSMZ5635, and *Neisseria cinerea* DSMZ4630 as representative members of an artificial lung consortium (see Table 1). These organisms represent the three most dominant phyla found in the human respiratory tract: *Pseudomonadota*, *Bacillota*, and *Actinomycetota*. *R. aeria*, *S. downei*, and *N. cinerea* are reference taxa in the expanded Human Oral Microbiome Database (eHOMD). Moreover, *P. koreensis* and *R. aeria* are included in the comprehensive human respiratory genome catalog. In addition, isolates of all four species have been reported in multiple studies as members of the oral and respiratory microbiota and have been detected in sputum, oropharyngeal swabs, and bronchoalveolar lavage fluid (BALF) from healthy individuals and patients with COPD or suspected respiratory infections (16–18). Furthermore, *R. aeria* and *P. koreensis* have been implicated in bronchitis and pneumonia in case reports (19, 20).

**TABLE 1:** Details of the artificial human lung consortium members

Selection criteria	*Pseudomonas koreensis*	*Rothia aeria*	*Streptococcus downei*	*Neisseria cinerea*
Phylum	*Pseudomonadota*	*Actinomycetota*	*Bacillota*	*Pseudomonadota*
Cultivation/biosafety	Biosafety level 1, (partially facultative) aerobic cultivation			
Respiratory tract association	Part of the non-pathogenic lung microbiome Can cause pneumonia	Part of the normal flora of the human oral cavity and the respiratory tract Can cause respiratory infection	Opportunistic respiratory pathogen	Transient low abundance commensal member of lung microbiome
Community shaping metabolic functions/traits (predicted)	Siderophore production Biosurfactant production Production of antimicrobial peptides	Carbohydrate fermentation	Biofilm production Fermentation	Quorum sensing
Relevance to consortium interplay	Potential nutrient competition Quorum sensing and biofilm production control	Potential metabolic complementarity pH management	pH management Metabolic complementarity Consortium protection through biofilm production	Community organization Competitive exclusion of pathogenic strains
References	(20–23)	(19, 24–26)	(27–29)	(30–32)

Although members of the *Bacteroidota* are also common in the lung, they were not included because they are frequently obligate anaerobes (33, 34) or require biosafety level 2 (BSL-2) conditions (35). All experiments were carried out in liquid free-cell cultures. This reflects the transient and healthy lung, where microbes inhabit thin, fluid-filled microenvironments shaped by ventilation and mucociliary clearance (6, 36). Under such conditions, microbes exist predominantly in planktonic or loosely attached states rather than well-established biofilms (37, 38). In contrast to the nutrient-limited conditions of a healthy lung, this study ultimately relied on a nutrient-rich cultivation medium. To better approximate physiological nutrient availability, we tested human cell culture media such as Dulbecco’s modified Eagle medium (DMEM) or Roswell Park Memorial Institute (RPMI) medium, as well as artificial sputum medium (ASM) (39). However, these conditions did not provide an adequate and stable environment for the growth of all bacterial strains. We therefore selected BHI medium, which is commonly used in studies of microbial interactions, despite this limiting the reproduction of the healthy lung environment (40–42).

### Understanding the growth behavior of mono- and co-cultures

**Fig 1:**
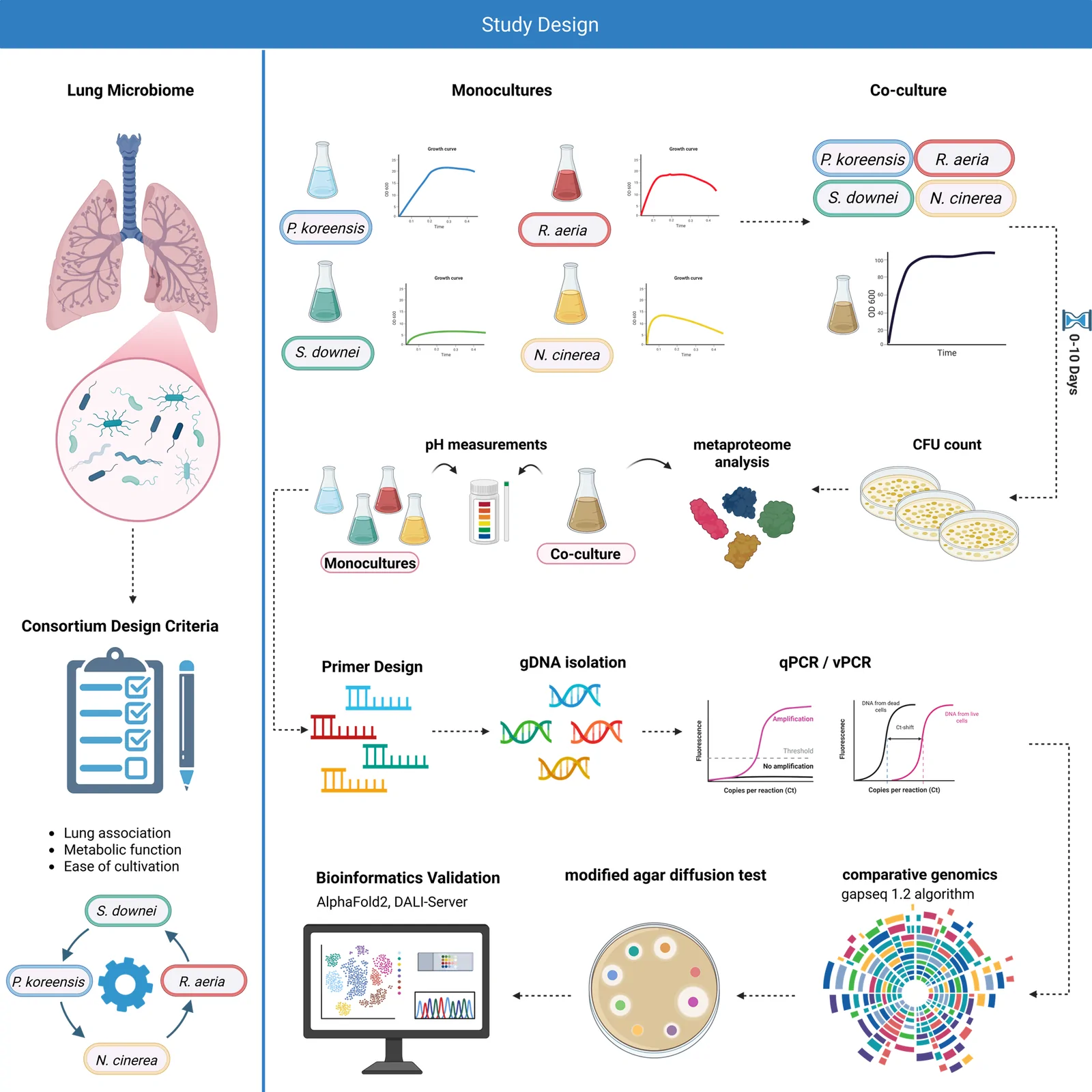
Schematic overview of the study design and experimental workflow. The design was conducted on comprehensive literature research combined with specific pre-established criteria in a bottom-up approach. To characterize the consortium, we investigated the growth behavior of monocultures compared to the co-culture using OD_600_ measurements, colony-forming unit (CFU) counts, and pH measurement, as well as quantitative and viability PCR over a time course of 10-day batch cultures. After 10 days of cultivation, a metaproteomic analysis of the cell pellet from the co-culture was carried out to examine functional protein expression. Using the gapseq algorithm, we conducted a comparative analysis of the co-culture’s genomes. Based on these results, we elaborated possible interactions using basic *in vitro* microbiological assays and bioinformatic tools such as AlphaFold2 and the DALI-Server. This figure was created using BioRender.

To investigate the microbe-microbe interaction and dynamics in the co-culture, we implemented an integrated experimental workflow. Initially, the growth characteristics of the selected species were assessed by monitoring microbial biomass, viable cell counts, and pH over 10 days. Subsequently, quantitative PCR (qPCR) and viability PCR (vPCR) were performed to determine the genomic composition of the co-culture. Finally, metaproteomic analysis was conducted after 10 days of cultivation to provide functional insights into the protein profiles (Fig. 1).

**Fig 2:**
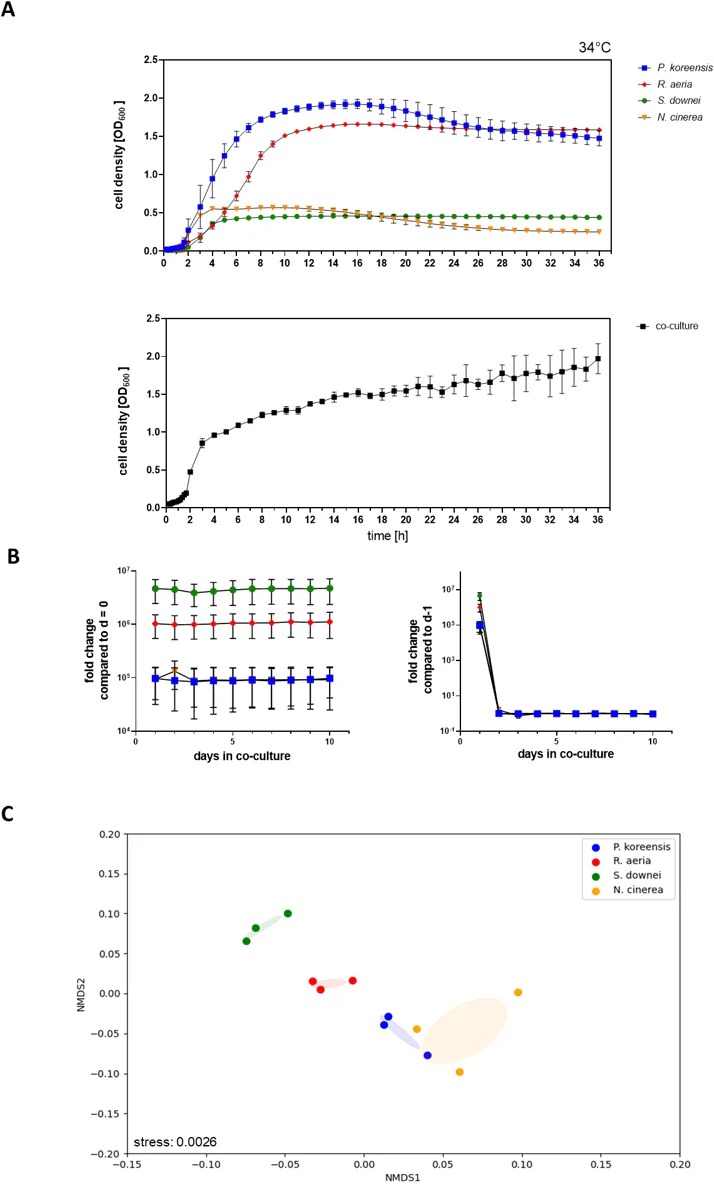
Growth behavior of *P. koreensis*, *R. aeria*, *S. downei*, and *N. cinerea* in mono- and co-culture. (**A**) To explore the different behavior of monocultures (upper graph) and the co-culture (lower graph) in brain heart infusion (BHI) medium, growth curves were recorded over a time period of 36 h at 34°C. Shown are the mean values of two individual experiments (*n* = 2), each performed in quadruplicate, ±SEM. Experimental setup can be found in Materials and Methods. (**B**) Fold change was calculated based on qPCR Ct values of the strains *P. koreensis*, *R. aeria*, *S. downei,* and *N. cinerea* in a batch co-culture compared to the respective previous day (right graph) and compared to the day of inoculation (left graph). The mean values from three independent co-culture experiments ± SEM, with two technical replicates each, are shown. (**C**) Reproducibility of the co-culture calculated by the Bray–Curtis dissimilarity index from qPCR Ct values of three individual co-culture experiments. The Bray–Curtis dissimilarity indices of the biological replicates are visualized as dots in the NMDS plot.

In monoculture, each strain exhibited distinct growth patterns over 36 h (Fig. 2A). For instance, *N. cinerea* reached a maximum OD_600_ of 0.57 ± 0.09 at 10 h and subsequently decreased by more than 50% to 0.25 ± 0.03 by 36 h. *P. koreensis* peaked higher at an OD_600_ of 1.92 ± 0.02 after 15 h and decreased roughly 25% to 1.48 ± 0.03 by 36 h. In contrast, *S. downei* grew only to an OD_600_ of 0.46 ± 0.01 at 15 h but then remained stable at 0.44 ± 0.003 by 36 h. *R. aeria* also retained culture density over time, with a peak OD_600_ of 1.66 ± 0.004 at 17 h and a slight decline to 1.59 ± 0.01 by 36 h. We then set up a co-culture with all four strains in BHI medium. Notably, the co-culture displayed increasing optical density throughout the 36-hour period, reaching a maximum OD_600_ of 1.97 ± 0.90 at 36 h (Fig. 2B). Statistical analysis using repeated-measures two-way ANOVA revealed a significant difference in growth trajectories over the 36-hour culture period between the monocultures and co-culture (*P* < 0.0001; see Data S1). The sustained growth in the co-culture suggests synergistic interactions among the strains. To get a more detailed understanding of the behavior of individual strains within the co-culture, we conducted quantitative PCR (qPCR) analysis targeting strain-specific sequences. The abundance of genomic DNA was determined by comparing the Ct values of each strain to those at inoculation (day 0) or on the previous day (day −1) and analyzed over the 10-day cultivation period (Fig. 2B). Between day 0 and day 1 of the co-culture, all strains exhibited a substantial increase in genomic abundance. *S. downei* showed the highest increase (4.67 × 10^6^-fold), followed by *R. aeria* (1.03 × 10^6^-fold). *P. koreensis* and *N. cinerea* displayed similar increases in genomic abundance, with fold changes of 9.70 × 10^4^ and 9.43 × 10^4^, respectively. The particularly pronounced initial growth of *S. downei* may be attributed to the rapid fermentative metabolism, typical for the genus *Streptococcus* (43). After this initial surge, the genomic copy numbers of all strains remained relatively stable throughout the cultivation period, suggesting that the co-culture maintained a steady-state microbial community over time. Next, we evaluated the reproducibility of the co-culture model by analyzing qPCR data from three independent experiments. Bray–Curtis dissimilarity indices (BCDI) were calculated based on Ct values for each strain, and non-metric multidimensional scaling (NMDS) was used to visualize community structure differences (Fig. 2C). One-way ANOVA of the BCDI values revealed no significant differences between biological replicates, indicating that the composition of the co-culture remained consistent across experiments (with *F* and *P* values reported in Data S4). The tight clustering of replicates in the NMDS plot (stress value below 0.0026) underscored the model’s reproducibility and reliability for studying microbial interactions in the lung microbiome.

### Co-culture demonstrates beneficial interactions and viability shifts

**Fig 3:**
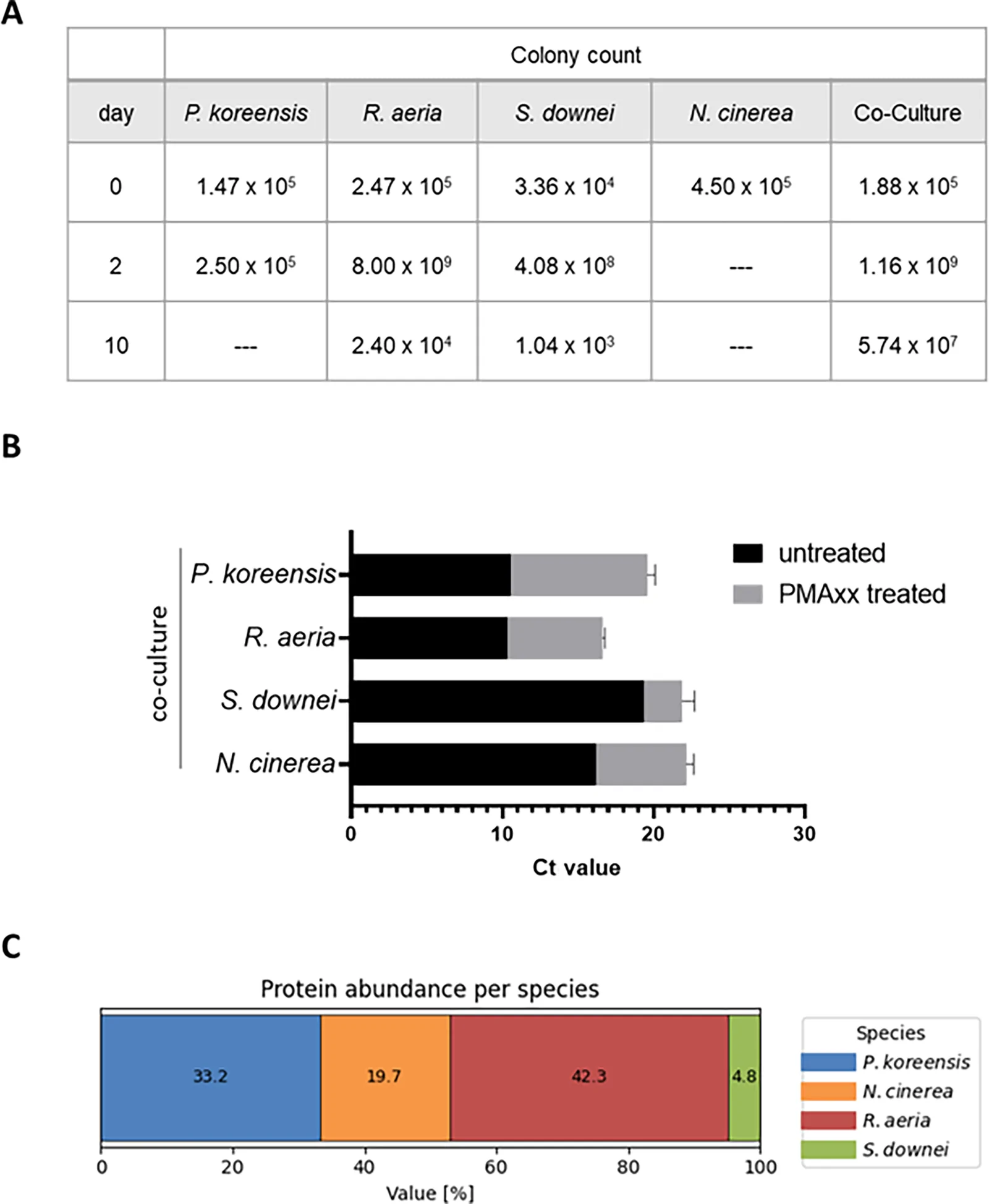
Investigation of growth dynamic changes of the artificial consortium compared to monocultures using colony-forming unit (CFU) counts, quantitative PCR, viability PCR, and pH measurement. Experimental setup can be found in Materials and Methods. (**A**) CFUs from mono- and co-cultures were counted from one representative experiment performed on 7 February 2024 (**B**) Viability PCR analysis of co-culture samples. Data from cultivation day 10 ± SEM are shown. (**C**) Stacked bar plot showing the relative abundance of proteins from the four bacterial species in the co-culture cell pellet after 10 days of batch cultivation. The *x*-axis shows the normalized abundance as a percentage. Each bar segment represents a different species. This visualization highlights the comparative contribution of each species to the overall protein abundance.

To determine the cell viability in both monoculture and co-culture, samples were collected on days 0, 2, and 10, and colony-forming units (CFUs) were counted (Fig. 3A). In monocultures, *N. cinerea* ceased viability by day 2, after an initial CFU count of 4.5 × 10^5^ on day 0. *P. koreensis* showed an increase in viable cells from day 0 (1.47 × 10^5^ CFUs) to day 2 (2.50 × 10^5^ CFUs) but produced no detectable CFUs by day 10. Conversely, *R. aeria* and *S. downei* maintained at least some viability by day 10, with CFU counts of 1.04 × 10^3^ and 2.40 × 10^4^, respectively. In the co-culture, however, the total CFU count remained high, with 5.74 × 10^7^ on day 10, suggesting that the consortium supports sustained viability of its members. Visual inspection of the plates revealed diverse colony morphology, which indicates the retention of complexity within the consortium. To further explore the composition of the co-culture, we performed viability PCR (vPCR) using PMAxx, a dye that does not permeate live cells but binds to free DNA from dead cells and thus prevents its amplification. As expected, the addition of PMAxx resulted in a general increase in Ct values in comparison to our qPCR data. But most intriguingly, the vPCR analysis revealed viable cells of all four strains in the co-culture even at day 10 (Fig. 3B). *R. aeria* exhibited the lowest Ct value (16.49), indicating that it was the dominant viable strain. *P. koreensis* followed with a Ct value of 20.11, whereas *S. downei* and *N. cinerea* had Ct values of 23.07 and 22.66, respectively. Taken together, our data from qPCR, vPCR, and colony count show that all bacterial strains of the co-culture exhibited rapid initial growth, particularly *S. downei*. However, the long-term viability varied significantly. *R. aeria* maintained both high abundance and viability, suggesting it as a dominant strain in the co-culture. In contrast, *S. downei* saw a reduction in viable cells over time from the early high levels. *P. koreensis* and *N. cinerea*, though struggling in monoculture over the long term, persisted in the co-culture, presumably through beneficial microbial interactions.

Next, the community composition was assessed by metaproteomic analysis using protein abundance as a proxy for biomass representation (44). This analysis yielded 49,985 unique peptides, which were assigned to 6,237 protein groups across the four species present in the consortium (Data S3). The most abundant proteins originated from *R. aeria* (42.3%, 1,783 protein groups), followed by *P. koreensis* (33.2%, 2,417 protein groups), *N. cinerea* (19.7%, 1,200 protein groups), and *S. downei* (4.8%, 800 protein groups). These relative abundances largely agreed with the composition of the consortium derived from vPCR analysis, supporting the consistency between genotypic and proteomic assessments (Fig. 3B and C). However, since we focused on endpoint metaproteomic analysis after 10 days of cultivation to obtain a comprehensive overview of the functional state of the established community, future work, including time-resolved metaproteomic and vPCR analysis of the community, could further enhance our understanding of the temporal development of metabolic interactions and community structure.

### Pronounced pH elevation in co-culture indicates interplay of acid-base metabolic processes

**Fig 4:**
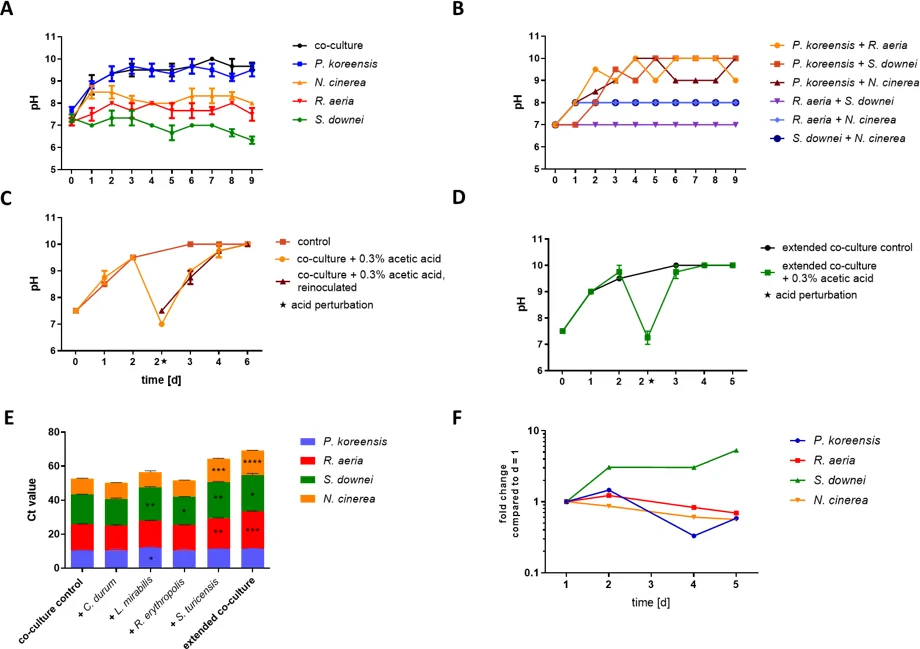
The pH dynamics in mono-, bi-, and co-cultures and the resilience of the four-strain consortium to an acetic acid pulse disturbance. Experimental set-up can be found in Materials and Methods. (**A**) pH was monitored during 10 days of cultivation for monocultures of *P. koreensis*, *R. aeria*, *S. downei*, and *N. cinerea* and for the four-strain co-culture (*n* = 3). Data are shown as mean ± SEM. (**B**) To assess the impact of community composition, pH trajectories of bi-cultures were recorded. Shown are mean values from one representative experiment performed on 17 April 2025. (**C**) To evaluate resilience and resistance to acid disturbance, the co-culture was supplemented after 2 days with 0.3% acetic acid. Data are shown as mean ± SEM (*n* = 2). (**D**) Resilience and resistance were then re-evaluated in an extended co-culture that contains four additional strains: *C. durum*, *L. mirabilis*, *R. erythropolis,* and *S. turicensis*. (**E**) qPCR evaluation of co-culture behavior after the addition of the new strains, *C. durum, L. mirabilis*, *R. erythropolis,* and *S. turicensis,* each individually and as a fully extended co-culture including the four original and the four additional strains. Data are shown as mean ± SEM after co-cultivation for 36 h (*n* = 2). The composition of the control artificial consortium was compared with (i) co-cultures containing each newly added strain and (ii) the fully extended co-culture. Statistical significance was defined as *P* ≤ 0.05 (*), *P* ≤ 0.01 (**), *P* ≤ 0.001 (***), and *P* ≤ 0.0001 (****). (**F**) qPCR assessment of the original co-culture strains *P. koreensis*, *R. aeria*, *S. downei*, and *N. cinerea* after 5 days of co-cultivation with the additional strains *C. durum*, *L. mirabilis*, *R. erythropolis*, and *S. turicensis*. Data show mean values from one representative experiment on 14 July 2025.

To evaluate the impact of metabolic activity on culture conditions, we monitored the pH of monocultures and the co-culture at the start and the end of the cultivation period. All cultures initially exhibited a pH of 7.3. After 10 days, significant pH changes were observed (Fig. 4A). The pH of the *S. downei* monoculture decreased to 6.3. This is not unexpected, as streptococci are known to ferment carbohydrates and produce lactic acid, contributing to acidifications (45). In contrast, the pH increased in the other monocultures, especially in cultures of *P. koreensis* (pH 9.5), but also in those of *R. aeria* (pH 7.5) and *N. cinerea* (pH 8.0). Interestingly, the co-culture showed the most pronounced increase, with a pH of 9.7 after 10 days of cultivation. The alkalization in the co-culture suggests that metabolic processes by certain strains may neutralize organic acids produced by others, e.g., by amino acid deamination (46, 47). One could assume that *P. koreensis* is the driving force in this process, with a pH of 9.5 in monoculture and high abundance in the co-culture. This interplay could facilitate the stability and viability of the consortium by maintaining pH within a range tolerable for all members, since growth of the genus *Neisseria* is known to be inhibited in lower pH environments (48).

To investigate how the community composition shapes the pH, the four strains were compared across pairwise bi-cultures (Fig. 4B). All bi-cultures containing *P. koreensis* shifted rapidly to alkaline conditions, reaching a basic pH of 9 to 10 within 3 days and maintaining this range over a 10-day observation period. In contrast, the bi-culture of *R. aeria* and *S. downei* remained stable at a neutral pH of 7.0 after inoculation. The co-cultivation of *R. aeria* with *N. cinerea* and *S. downei* with *N. cinerea* converged after 1 day to a pH of 8.0 and remained constant over 10 days. This indicates that *P. koreensis* is indeed a key contributor to the alkaline shift in the mixed community. A mechanistic explanation could be the production of basic biogenic amines, which is widespread within the genus *Pseudomonas* (49). This could drive alkalinization in mono- and co-culture and buffer the community upon acid exposure.

### Co-culture shows robust resilience to acid perturbation and community expansion

To test whether the four-member consortium is resilient to acid perturbation, an established co-culture was challenged with 0.3% (vol/vol) acetic acid, resulting in a reduction of the pH to 7 (Fig. 4C). Airway acidity is associated with multiple lung diseases and inflammatory markers because it shifts the system toward the physiological airway pH range (approximately 7–8), whereas several disease states have been linked to values below 6.5 (50). Notably, acetic acid is typically used in *in vitro* inflammation models to induce inflammatory processes. It has also been reported to alter the oral microbiome and is associated with less diverse microbiome composition (51). To distinguish recovery driven by community activity from potential medium effects, an aliquot of the acid-treated culture was transferred into fresh medium 2 h after perturbation, ensuring both conditions experienced the same initial disturbance. Using the pH time series (Fig. 4C and D), resistance (RS) and resilience (RL) were calculated. Resistance captures the magnitude of the immediate deviation following disturbance, and exposure to 0.3% acetic acid caused a ~2.5 unit drop in pH (9.5 to 7.0), indicating limited buffering capacity against the initial acidification. Resilience captures the rate of recovery, and in both the culture maintained in acidified medium and the reinoculated culture, the pH recovered to approximately 8.5 within 24 h (t3) and returned to the untreated control range (pH 9–10) after an additional 24 h (t4), indicating rapid and complete restoration of the pre-disturbance alkaline state within 2 days. The similar recovery trajectories in both conditions suggest that pH restoration is not solely attributable to medium exchange, but reflects a robust community-level capacity to re-establish alkaline conditions. Viability measurements using CFU counts supported that the community remained viable complex after the perturbation. CFU/mL increased from 8.75 × 10^5^ at inoculation (t0) to 2 × 10^9^ at 24 h after acid addition (t3), confirming the presence of living cells in the treated co-culture after the acetic acid challenge. CFU counts reflect overall viability and do not resolve the abundance of each member. Thus, our data support functional rather than compositional resilience. To test whether the established consortium remains stable within a larger community context, the co-culture model was challenged with different lung-associated strains: *Rhodococcus erythropolis* DSMZ43066*, Corynebacterium durum* DSMZ45333, *Lautropia mirabilis* DSMZ11362, and *Siccibacter turicensis* DSMZ18397, selected as aerobic BSL-1 type strains from the human respiratory genome catalog (18). The addition of *R. erythropolis*, *C. durum*, or *L. mirabilis* did not result in major changes in the growth kinetics, with maximum OD values of approximately 1.4–1.6 after ~30 h and doubling times of around 3.5 h. In contrast, when supplementing the established co-culture with *S. turicensis* or all four new strains combined, higher maximum OD_600_ values of 1.7 and faster doubling times of 1.6 h and 2.2 h, respectively, were recorded. Interestingly, all extended co-cultures showed stable or increasing OD_600_ values over the 36-hour period (Fig. S2), suggesting stabilizing interactions in the community setting. Strain-specific effects were assessed by qPCR, comparing Ct values of the original consortium members across the extended conditions relative to the original co-culture (Fig. 4E). The challenge with *L. mirabilis* resulted in the apparent growth suppression of *P. koreensis* and *S. downei,* with significantly higher Ct values for those two strains (+1.8 and +2.3 respectively). A similar picture was obtained for *R. aeria*, *S. downei*, and *N. cinerea* when challenged with *S. turicensis*, giving significantly higher Ct values of +2.5, +3.7, and +4.3 respectively. This could be explained by the high growth rates that *S. turicensis* can achieve under these cultivation conditions (Fig. S2A and B), resulting in an increasingly difficult environment for the original consortium due to nutrient competition. Nevertheless, the original strains were clearly detectable in any of the challenged co-cultures, with Ct values between 13 and 21, and their relative composition remained stable. This could be confirmed by a 5-day co-culture of the original consortium with all four new strains, which showed only minor changes in relative DNA abundance (Fig. 4F). Interestingly, perturbation of this fully extended co-culture showed the same resistance to the pH drop (RS = 0.58) but a faster recovery within 1 day toward the initial pH (RL = 1) (Fig. 4D). Overall, the original consortium maintained relatively uniform and stable growth when challenged with different stress factors and demonstrated remarkable compositional resilience.

### Comparative genomics identifies shared and unique pathways in the microbial consortium

**Fig 5:**
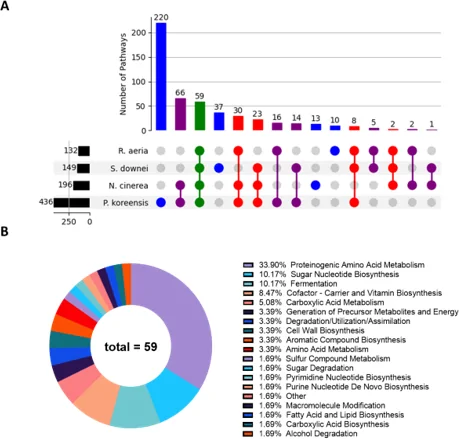
Metabolic potential of the artificial consortium using the genomic scale model gapseq. In order to compare the metabolic potential of the four strains *P. koreensis*, *R. aeria*, *S. downei,* and *N. cinerea*, pathways based on the MetaCyc database were predicted using gapseq v1.2. (**A**) These pathways were then matched with each other, and unique and shared pathways were separated in an UpsetPlot. The colors of the connected dots indicate unique pathways (blue), pathways shared by only two strains (purple), pathways shared by three strains (red), and pathways that occur in all strains (green). (**B**) The pathways were further classified into superclasses using the MetaCyc classification; shared pathways were illustrated as pie charts.

**Fig 6:**
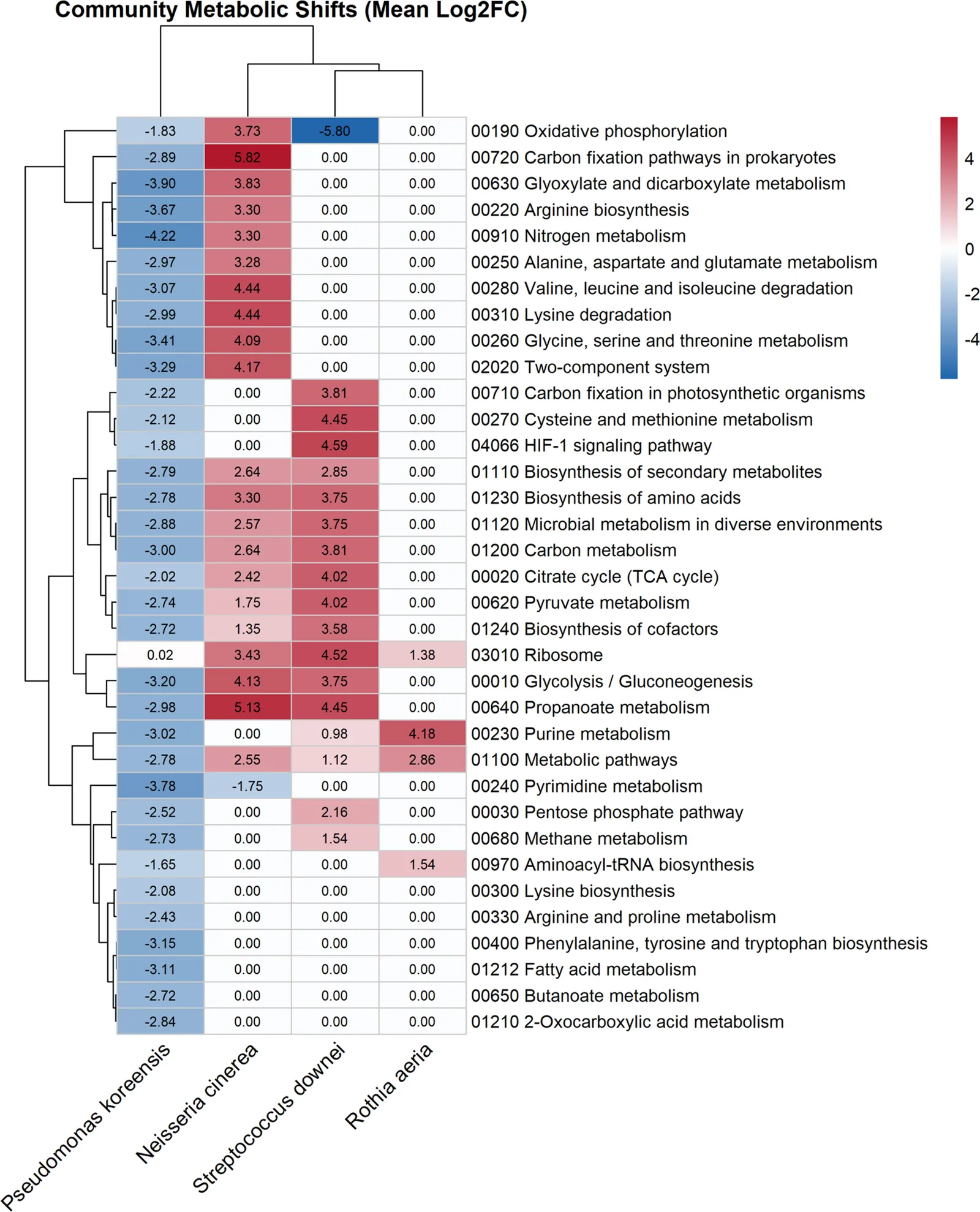
Functional adaptation from monoculture to community cultivation. The change in pathway abundance from monoculture to community culture is shown for all four species, and a blue-to-red color code is used to indicate the extent and direction of the changes.

To evaluate the metabolic capacities of each strain and possible interactions inside the model community, we conducted genomic-based pathway predictions using the gapseq algorithm (v1.2). Such pathways may contribute to ecological niche separation by supporting synergistic, cooperative, or antagonistic dynamics within the co-culture. Following the evaluation of metabolic pathways for each bacterial strain, we identified both unique pathways and those shared by multiple strains (Fig. 5B and Fig. 6A). A library of all pathway files is given in Data S5. A total of 436 metabolic pathways were predicted for *P. koreensis*, 132 for *R. aeria*, 149 for *S. downei*, and 196 for *N. cinerea*. Of these, 59 pathways were shared across all four strains and were primarily involved in essential metabolic processes, including proteinogenic amino acid synthesis (44%) and sugar nucleotide biosynthesis (11%), as well as cofactor, carrier, and vitamin biosynthesis (Fig. 5B). The presence of fermentation pathways (10%) suggests potential cooperation among the strains, enabling the consortium to maintain metabolic stability. The redundancy of metabolic pathways might also reflect the resilience to environmental influences, as multiple strains can fulfill similar functions within the group.

*P. koreensis* stands out with 220 predicted unique metabolic pathways, reflecting its extensive metabolic versatility, characteristic of the *Pseudomonas* genus (52–57). They likely enable *P. koreensis* to thrive in diverse and challenging ecological niches. In our lung microbiome model, one possible strategy for interference competition involves the secretion of antimicrobial molecules (58). Notably, the gapseq algorithm identified two putative pathways for the synthesis of antibiotics in *P. koreensis*, i.e., for gramicidin- and for polymyxin-like compounds. Gramicidin targets both gram-positive and gram-negative bacteria (59), whereas polymyxin A is mainly active against gram-negative bacteria (60). According to our analysis, *P. koreensis* also features pathways for the production of two siderophores similar to vanchrobactin and pyoverdine, respectively. Such molecules play crucial roles in iron acquisition, a vital process for both interspecies competition and host nutritional immunity (61, 62). In iron-limited environments such as the lung, where competition for trace metals is high, the production of siderophores facilitates iron uptake. However, this capacity may also support other consortium members through siderophore sharing. Biofilm formation is another small molecule-mediated competitive strategy. This process is facilitated by biosurfactants such as massetolide A and viscosin, with similar biosynthetic pathways predicted in *P. koreensis*. Surfactants reduce surface tension, promoting biofilm formation, which enhances survival in nutrient-limited environments and fosters interspecies communication (7, 63).

A total of 132 pathways were predicted in *R. aeria*, of which 10 were unique. Similar to *P. koreensis*, *R. aeria* carries a putative siderophore pathway, in this case annotated for yersiniabactin. However, a manual review of the *R. aeria* genome using antiSMASH did not recover all of the genes required for the biosynthesis of yersiniabactin (64). Instead, the putative biosynthetic gene cluster showed 77% similarity to an already described enterobactin biosynthetic gene cluster from *Rothia mucilaginosa* (65). Members of the genus *Rothia*, including *R. aeria*, have also been suggested to contribute to the microbial community by providing substrates for other bacteria, due to their ability to ferment complex carbohydrates and proteins (66). This aligns with our findings of pathways involved in amine (10%) and carboxylic acid degradation (10%) using gapseq.

The 37 unique pathways in *S. downei* include a pathway for the fermentation product butanediol. *In vitro* experiments have shown that butanediol production leads to a threefold upregulation of the transcriptional regulators LasI and LasR, which control quorum sensing via autoinducer synthesis and gene activation (67). Moreover, *Streptococcus* species are known to produce acidic fermentation byproducts in the oral microbiome, contributing to conditions such as dental caries (45). In our comparative genomics study, we identified corresponding fermentation pathways (13.33%) in *S. downei* and observed significant acidification in its culture. This may reflect its role in broader microbiome dynamics and influence the consortium by affecting pH levels.

Of the 13 unique pathways identified in *N. cinerea*, the pathway for autoinducer AI-2 stands out. AI-2 is a quorum sensing molecule synthesized by a wide range of gram-positive and gram-negative bacteria (68). It is considered a “universal” signaling molecule for cell-cell communication, allowing different species to coordinate activities such as biofilm formation, virulence factor production, and nutrient acquisition (69).

### Validation of genome-based metabolic modeling by proteomics

**Fig 7:**
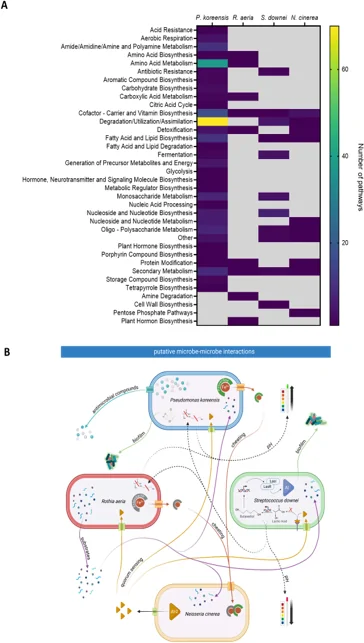
Putative consortium interactions based on pathway prediction. Pathways were predicted based on the MetaCyc database using the gapseq (1.2) algorithm. Unique pathways were organized as a heatmap (**A**). Putative interactions in the consortium based on the conducted comparative genomics and metaproteomic analysis were visualized using BioRender (**B**).

To validate the genome-based predictions, we performed proteomic analyses of monocultures and metaproteomic analysis from co-culture samples. In total, 4,929 protein groups were identified in the monocultures and 4,323 in the co-culture (Data S8A). Taxonomic assignment of the detected proteins resolved the species composition of the community and was in good agreement with species-specific qPCR results (see Fig. 3B and C). The proteomic data further confirmed a reduced relative abundance of *P. koreensis* in the community compared to monoculture, while *R. aeria* showed increased abundance under co-culture conditions. To investigate functional adaptations associated with the transition from monoculture to community growth, we compared protein occurrence between these conditions. The overlap was moderate; only 36% of proteins were detected in both monocultures and co-culture, whereas 33.8% were unique to monocultures and 30.2% were exclusively detected in the community (Data S8B). Notably, proteins assigned to *R. aeria* dominated both the monoculture-specific and community-specific fractions, whereas *P. koreensis* proteins were primarily found in the shared fraction. Species-resolved volcano plots of protein abundance showed that most significantly altered proteins originated from *P. koreensis*, with a general trend toward reduced abundance in the co-culture (Data S8C). In contrast, proteins from the other species were predominantly increased in the community, indicating distinct species-specific adaptation strategies. To further explore these differences, we analyzed significantly affected pathways (Fig. 7).

In *P. koreensis*, many pathways were reduced under co-culture conditions, particularly those related to amino acid metabolism, including arginine, nitrogen, glycine, serine, and glutamate metabolism. Interestingly, these same pathways were strongly increased in *N. cinerea* in the co-culture.

In *S. downei*, a broader range of metabolic pathways showed increased abundance, including core cellular functions, such as ribosome-associated processes, propanoate metabolism, and central carbon metabolism (e.g., citrate cycle and pyruvate metabolism). Additionally, cysteine and methionine metabolism and the HIF-1 signaling pathway were elevated. Both pathways are commonly associated with oxidative stress responses, which may be linked to environmental changes such as pH shifts occurring in co-culture. Despite being the dominant species in the community, *R. aeria* exhibited relatively few significantly altered pathways. The most notable change was an increase in purine metabolism.

Overall, these observations suggest that *P. koreensis* adapts to community conditions by downregulating multiple metabolic pathways, possibly reflecting reduced growth or increased availability of metabolites available in the shared environment. In contrast, the increased abundance of metabolic pathways in *N. cinerea* and *S. downei* may indicate either enhanced metabolic activity or the production of metabolites that are released into the environment. Given the relatively stable growth dynamics observed (Fig. 4), these species contribute metabolites that are subsequently utilized by *P. koreensis*.

#### Predicted antimicrobial pathways in *P. koreensis* do not manifest in co-culture

Following the pathway predictions from the comparative genomics analysis, we examined whether *P. koreensis* exhibits antagonistic behavior through the secretion of antimicrobial substances. To test this, a modified agar diffusion assay was performed. Possible inhibitory effects between *P. koreensis*, *R. aeria*, *S. downei*, and *N. cinerea* were assessed in different pairwise combinations. No inhibition zones were detected in any of the tested conditions, indicating that none of the strains exerted antimicrobial activity under this setup. This suggests that the strains do not produce antimicrobial substances at levels sufficient to inhibit neighboring species in agar-based assays. Metaproteomic analysis supports this interpretation, in which no proteins linked to known antimicrobial biosynthesis pathways were detected. Together with the observation that all strains could be co-cultured without antagonism, these findings point toward non-antagonistic interactions such as metabolic cooperation, resource sharing, or competitive exclusion without direct inhibition, as likely more influential in shaping the community structure.

At the same time, antimicrobial production cannot be completely excluded. It is possible that such compounds are produced only transiently during earlier stages of cultivation, such as initial colonization or niche establishment, and are subsequently downregulated by day 10 (70, 71). Furthermore, antimicrobial biosynthesis is energetically demanding, and its expression is often tightly regulated and suppressed in environments where microbial competition is minimal (72, 73).

#### *N. cinerea* may exemplify metabolic complementarity through exploitation of siderophores

Because *N. cinerea* ceased to grow in monoculture but persisted in co-culture; we hypothesize that it benefits from substances secreted by the other strains. One possible explanation is the limited availability of iron, which is often present as insoluble ferric iron (Fe³^+^) in oxygenated environments, making it inaccessible to many organisms (74). Some microorganisms overcome this limitation by producing siderophores, iron-chelating compounds that solubilize Fe³^+^, allowing them to survive in low-iron environments (61). Our pathway predictions revealed that siderophore biosynthetic pathways were present only in *P. koreensis* and *R. aeria*, with siderophore production being verified only in *P. koreensis* through our metaproteomic data. However, studies have shown that some microorganisms lacking siderophore-forming capabilities can produce receptors that enable them to utilize siderophores secreted by others. This phenomenon is known as “siderophore piracy” or “cheating” and has the potential to affect the dynamics of microbial communities (75–77).

To investigate the potential for siderophore-mediated interactions within the co-culture, we performed protein homology searches using the NCBI BLASTp tool to identify candidate proteins involved in siderophore uptake in *P. koreensis* and *N. cinerea*. Comparative genomic analysis revealed that a putative TonB-dependent siderophore receptor from *N. cinerea* (WP_167395505.1) shared the highest sequence similarity with a known TonB-dependent siderophore receptor from *P. koreensis* (WP_083367461.1), exhibiting 41% identity and 85% sequence coverage.

To further explore potential functional similarities, we predicted the three-dimensional structure of the *N. cinerea* receptor using AlphaFold2 (Data S6). Structural alignment via the DALI server revealed a strong resemblance to established siderophore receptors, such as FpvA, a ferripyoverdine receptor from *Pseudomonas aeruginosa*, with a Z-score of 48.3, indicating significant structural similarity. Supporting these *in silico* findings, we detected the predicted TonB-dependent siderophore receptor (D0W306, WP_167395505.1) in the metaproteome of *N. cinerea*. In addition, several TonB-dependent receptors associated with *Pseudomonas koreensis* (UniProt taxonomy ID: 198620) were identified, including A0AA94JF18, A0AA94ELX1, A0AA94EUG8, A0AA94JFD4, A0AA94ES30, siderophore-interacting proteins (A0AA94ERF0), ferric uptake regulators (A0AA94EML5, A0AA94ENA5), and A0AA94EPI8, a TonB-dependent copper receptor. This finding points toward the possibility of active siderophore-mediated iron acquisition by *P. koreensis* and possible siderophore-mediated “cheating” by *N. cinerea*, thereby enhancing its survival in the co-culture. Such metabolic complementarity could contribute to the observed stability and coexistence within the microbial consortium. While these results are compelling, experimental validation is required to confirm this cross-species interaction. Future experiments could include siderophore uptake assays, growth experiments of *N. cinerea* in iron-limited media supplemented with defined siderophores, or the construction of siderophore-deficient *P. koreensis* mutants to assess their effect on *N. cinerea* proliferation.

#### Metabolic cross-feeding may explain alkalinization of the consortium

Although *S. downei* showed an acidifying phenotype in monoculture, the co-culture reached a final pH of 10, indicating that community-level metabolism overweighted its fermentative output. The absence of canonical biofilm-associated markers such as massetolide A and viscosin in the proteomic data set further suggests that these interactions do not rely on stable surface-associated biofilm formation. Instead, the consortium appears to depend on soluble signaling (i.e., AI-2) and metabolic cross-feeding, pointing to a more dynamic mode of interaction (78).

Metaproteomic data revealed that *S. downei* maintains a fermentative profile in co-culture, characterized by the increases in L-lactate dehydrogenase (A0A380JEB6) and multiple glycolytic enzymes, including glyceraldehyde-3-phosphate dehydrogenase (A0A380JF47), phosphoglycerate kinase (A0A380JE11), enolase (A0A380JFF2), and pyruvate kinase (A0A380JFU5) in co-culture. However, the strongly alkaline endpoint of the co-culture indicated that this acidifying potential is counterbalanced and ultimately surpassed by the metabolic activities of the other community members.

Consistent with this, the co-cultured species exhibited functions that are complementary to acid scavenging and alkalinization. *N. cinerea* showed increased levels of pyruvate dehydrogenase components (D0W0E4, D0W0E3; D0W0E2), suggesting a shift toward oxidative central carbon metabolism and enhanced pyruvate assimilation in co-culture. By scavenging pyruvate before it can be channeled into fermentation, *N. cinerea* shunts carbon away from lactate production, thereby mitigating the acidifying output of *S. downei*. Complementing this reduction in organic acid precursors, the co-culture proteomic profile of *P. koreensis* revealed an enrichment of enzymes involved in the alkalinization of nitrogen metabolism, such as arginine deiminase (A0AA94JJ53), ornithine carbamoyl-transferase (A0AA94JIV7), carbamate kinase (A0AA94JKD8), and urease subunits (A0AA94EPQ6, A0AA94EQC8), which could contribute to the release of ammonia and the partial neutralization or alkalinization of the medium.

*R. aeria* appears to have contributed to lactate turnover. The proteome included L- and D-lactate dehydrogenase (A0A2Z5R0E4, A0A2Z5R113) as well as additional proteins annotated as predicted L-lactate dehydrogenases (A0A2Z5QXU4, A0A2Z5QY56, A0A2Z5QXI2), indicating an expanded capacity for lactate utilization. These findings support a model in which *S. downei* provides fermentative acids, while the other members consume these products and activate pathways that shift the overall balance toward alkalinity.

Evidence of coordinated behavior was also observed at the level of cell-cell signaling. Although LasR and LasI transcriptional regulators were not detected, the presence of the AI-2 pathway enzyme LuxS in *R. aeria* (A0A2Z5QYK0) and *N. cinerea* (D0W421) suggests that alternative quorum-sensing systems may be active within the consortium, contributing to coordinated gene regulation. Previous studies have shown that quorum sensing can trigger a metabolic switch from producing lactic and formic acid to butanediol, thereby preventing excessive acidification (79). In line with this, we detected key enzymes involved in butanediol metabolism, including 2,3-butanediol dehydrogenase (A0A380JDI2) in *R. aeria* and diacetyl reductase (A0A380JDP7) and acetoin utilization protein (A0A380JDP7) in *S. downei*.

Together, the alkaline endpoint of the co-culture does not contradict the fermentative nature of *S. downei* but rather reflects a coordinated community response involving acid production, acid consumption, alkalinizing nitrogen metabolism, and alternative quorum sensing, which collectively stabilize the consortium (Fig. 6B). Such metabolic shift may also be relevant in the context of inflammatory respiratory diseases (80).

### Conclusion

Simplified models are valuable tools for unraveling the interactions and behaviors of complex microbial communities. In this study, we established a novel artificial lung microbiome consisting of *Pseudomonas koreensis*, *Rothia aeria*, *Streptococcus downei*, and *Neisseria cinerea*. This consortium showed stable and reproducible growth over a 10-day batch co-culture period in brain heart infusion medium. Comparative genomics revealed that consortium members possess both unique and shared metabolic pathways, facilitating intricate interspecies interactions. By integrating these bioinformatic analyses with *in vitro* microbiological studies and metaproteomic analyses, we were able to identify key features of the microbial consortium, such as metabolic complementarity and siderophore-mediated “cheating” mechanisms. Specifically, siderophore production enhances iron acquisition from the respiratory environment, supporting both individual bacteria and the consortium as a whole (81). Furthermore, the degradation of acidic metabolites protects pH-sensitive species and maintains the pH balance of the lung epithelium, which is essential for adequate mucociliary clearance and innate immune defense mechanisms (82). Collectively, these findings support the notion that a functional lung microbiome is defined by the fulfillment of key functions rather than by the mere presence of specific strains, making this model a useful framework for further studies. Notably, the ecological principles captured by this consortium have previously been described mainly for pathogenic respiratory bacteria, demonstrating that these processes equally shape the non-pathogenic taxa that dominate healthy airways and thereby underscoring the broader validity of the model. Beyond its conceptual value, this study provides a foundation for investigating the human lung microbiome in a controlled setting, opening avenues to explore interspecies communication, microbial responses to external perturbations, and pharmacological interactions, including both the impact of drugs on the microbiome and the reciprocal influence of the microbiome on drug responses.

## MATERIALS AND METHODS

### Maintenance of bacterial cultures

The consortium consists of the following four bacteria: *Pseudomonas koreensis* (DSMZ 16610), *Rothia aeria* (DSMZ 14556), *Streptococcus downei* (DSMZ 5635), and *Neisseria cinerea* (DSMZ 4630). Bacterial strains used for compositional resilience testing were *Corynebacterium durum* (DSM 45333), *Siccibacter turicensis* (DSM 18397)*, Lautropia mirabilis* (DSM 11362), and *Rhodococcus erythropolis* (DSM 43066). The bacterial strains were cultured in brain heart infusion (BHI) medium at 34°C. Glycerol stock solutions were prepared from all strains after 16-hour overnight culture, which were stored at −80°C and used for further experiments.

In contrast to the nutrient-limited conditions of a healthy lung, this study ultimately relied on a nutrient-rich cultivation medium. We therefore tested alternative media to better approximate physiological nutrient availability, such as Dulbecco’s modified Eagle medium (DMEM) or Roswell Park Memorial Institute (RPMI) medium, as well as artificial sputum medium (ASM) (39). However, none of these conditions supported stable growth of all bacterial strains. Consequently, brain heart infusion (BHI) medium was used as it provides stable growth and is commonly applied in studies of microbial interactions (40–42).

### Growth curves

To record the growth curves, the optical density (OD) at 600 nm was measured with a plate reader (Cytation 1, Agilent) over a time period of 36 h. For this purpose, overnight cultures (*t* = 24 h) of each strain were inoculated in BHI at 34°C. The next day, OD_600_ was determined by spectrophotometer (Evolution 201, Thermo Scientific), and the respective monocultures were inoculated into a 24-well plate (Sarstedt) to an OD_600_ of 0.05. The final volume was 1 mL. The plate was then incubated in the plate reader at 34°C with fast orbital shaking, and OD_600_ was recorded over a period of 36 h. This experiment included three biological replicates with each four technical replicates. As *R. erythropolis* shows strong aggregation and dilution of the cultures is not possible during automated measurements in the plate reader, additional manual measurements are performed using a spectrophotometer. In these measurements, the samples are diluted for each reading in order to calculate the actual OD afterward. For this, one shake flask per bacterial strain is inoculated in 25 mL BHI medium to an OD of 0.05. OD_600_ is determined hourly using a spectrophotometer (Evolution 201, Thermo Scientific), and the volume of medium required for each measurement (between 100 and 1000 µL) is replaced with fresh medium.

### Inoculation of the co-culture

Fresh overnight cultures of *P. koreensis*, *R. aeria*, *S. downei*, and *N. cinerea* were incubated at 34°C for 16 h in BHI medium prior to inoculation of the co-culture. Cell counts were then performed using a Bürker-Türk counting chamber, and solutions containing a concentration of 1 × 10^7^ cells/mL for each strain were prepared. Subsequently, 1 mL of each cell suspension was inoculated into 46 mL of fresh BHI medium. Addition of the new strains was performed in the same manner, using 1 × 10^7^ cells/mL in a total volume of 42 mL BHI. Following the inoculation of the co-culture, samples were collected for day 0 after 10 min to guarantee thorough mixing of the cell suspensions. Subsequent samples were gathered on each consecutive day thereafter. For sampling, 900 µL of cell suspension was substituted with fresh medium. Depending on the subsequent experiment, the sample was (i) directly incubated with the viability dye PMAxx for vPCR, (ii) centrifuged, with supernatant discarded, the pellet was washed twice with phosphate-buffered saline, and finally stored at −20°C for qPCR experiments, or (iii) serially diluted and plated on BHI plates to count colonies.

### Primer design

The primers for qPCR (Table 2) were designed for each strain using Benchling 2023 (retrieved from https://benchling.com) and commercially synthesized by Eurofins Genomics. They possess the same copy number across species and were tested for cross-reactions using standard PCR (BioRad CFX96 Deep Well C1000 Thermal Cycler).

**TABLE 2:** Primers used for qPCR and vPCR to observe co-culture behavior on a genomic level

Strain	Gene		Sequence	Melting temp (°C)	Product size (bp)	Reference
*P. koreensis*	tuf	fw	ACG GTA GCC TTT GAA GAA CG	63	89	(83)
		rv	TTA AGC CGC ACA CCA AGT TC			
*R. aeria*	ctg1_ 792	fw	CAT TCG CCA GTA CAC ACA ATC C		123	–
		rv	ACA CCC ATC GTC CAC AGT TC			
*S. downei*	gtfl	fw	GAA ACC AAC CCA ACT TTA GCT TGG AT		329	(84)
		rv	ATG GAG TGA TTT TCC ATC GGT ACT TG			
*N. cinerea*	ctg1_ 1484	fw	GCC GGA CAT GAT GTG GAA AC		87	–
		rv	GAG TTC GAG CAA ATC CCA AAC G			

### gDNA extraction and qPCR

DNA extraction from the frozen cell pellets was performed according to the Monarch DNA Isolation Kit protocol. The extracted genomic DNA (gDNA) was stored at −20°C until qPCR analysis. Preparation of the master mix and loading of the qPCR plate were conducted under sterile conditions. Prior to work commencement, the laminar air flow bench and all equipment, including the qPCR plate, were treated with DNA-ZAP to eliminate any potential contaminating DNA. The qPCR plate was subsequently rinsed with sterile water and allowed to air-dry under the bench. Meanwhile, master mixes were prepared for each strain individually. The CAPITAL qPCR Green Master Mix LROX (biotechrabbit) was utilized for this purpose, with primers added at a concentration of 0.3 µM. Following plate loading with the master mix, 6 µL of water, instead of template, was added to each well for negative controls, and the wells were sealed. The plate was then removed from the bench, and the isolated gDNA (60 ng/µL) was added to the corresponding wells. Finally, the plate was sealed with a transparent foil and inserted into the Bio-Rad qPCR cycler. Each sample and no-template control were measured in duplicate. Fold change was then calculated based on the obtained Ct values for each strain at each time point by first calculating delta Ct by: ΔCt = Ct_*t* = *x*_ − Ct_*t* = 0_. The fold change in comparison to the day of inoculation could then be calculated as: 2^(−ΔCt)^. This experiment included three biological replicates with two technical replicates each.

### Viability PCR

In order to differentiate live from dead cells in the co-culture after 10 days, we performed a viability PCR using the PMAxx dye. For this, 400 µL samples were treated with 1 µL of a 10 mM PMA stock solution and incubated at room temperature for 10 min in the dark under shaking. The samples were then put under a light source (Osram L 18W/880 skywhite) to ensure cross-linking of the dye with the free DNA for 15 min. After centrifugation at 5,000 × *g* for 10 min, the samples were then used for DNA extraction using the Monarch DNA Isolation Kit protocol, and qPCR was performed according to “gDNA extraction and qPCR” above.

### Metaproteomic sample preparation

For metaproteomic analysis, cell pellets from 10-day co-culture batch incubations (*n* = 3 biological replicates) were used. Each sample underwent three freeze-thaw cycles in liquid nitrogen to promote cell lysis, followed by resuspension in lysis buffer containing 8 M urea, 2 M thiourea, and 1 mM PMSF. Bacterial cells were mechanically disrupted using bead beating (FastPrep-24, MP Biomedicals, Santa Ana, CA, USA; 5.5 ms, 1 min, three cycles) and subsequently subjected to ultrasonication (UP50H, Hielscher, Teltow, Germany; cycle 0.5, amplitude 60%). Lysates were clarified by centrifugation (10,000 × *g*, 10 min), and the supernatant was collected for protein quantification using the Bradford assay. Protein clean-up and enzymatic digestion were carried out using a modified filter-aided sample preparation (FASP) protocol. Vivacon 500 centrifugal filter units (Sartorius, Göttingen, Germany; 10 kDa MWCO) were pre-equilibrated with UT buffer (8 M urea, 2 M thiourea in water) and centrifuged at 14,000  ×  *g* for 20 min at room temperature. All subsequent centrifugation steps were conducted under the same conditions. A total of 100 µg of protein lysate was loaded onto each filter unit and washed twice with UA buffer (8 M urea in 0.1 M Tris-HCl, pH 8.5). Disulfide bonds were reduced by incubation with 200 µL of 10 mM dithiothreitol (DTT) in UA buffer at 37°C without shaking. Proteins were then alkylated in the dark with 200 µL of 50 mM iodoacetamide (IAA) in UA buffer at room temperature for 10 min. Following centrifugation, protein alkylation and digestion were completed as previously described. Resulting peptides were reconstituted in 0.1% formic acid before mass spectrometric analysis. For LC-MS/MS analysis, 1 µL of peptide solution was injected into a Vanquish Neo nano-HPLC system (Thermo Fisher Scientific). Peptides were first trapped on a C18 trapping column (Acclaim PepMap 100, 3 µm, 75 µm × 2  cm, Thermo Fisher Scientific) using a mobile phase of 98% water, 2% acetonitrile (ACN), and 0.5% trifluoroacetic acid (TFA) at a flow rate of 5 µL/min. Peptide separation was performed on an analytical C18 column (PepMap 100, 3 µm, 75 µm × 15  cm) with a flow rate of 300 nL/min. The mobile phases consisted of 0.1% formic acid in water (solvent A) and 80% ACN with 0.08% formic acid (solvent B). Peptides were analyzed on an Orbitrap Exploris 480 mass spectrometer (Thermo Fisher Scientific) operating in data-dependent acquisition (DDA) mode. Full MS spectra were acquired at a resolution of 60,000 (at 200 *m*/*z*) with an automatic gain control (AGC) target of 3  ×  10⁶ ions. MS/MS fragmentation was performed using higher-energy collisional dissociation (HCD). Raw mass spectrometric data were processed using Proteome Discoverer (v2.5.0.400, Thermo Fisher Scientific). Protein identification was performed using the Sequest HT search engine against the proteomes of the four co-cultured species—*P. koreensis* (5,799 entries), *N. cinerea* (2,126 entries), *Rothia aeria* (2,357 entries), and *S. downei* (2,055 entries) (UniProt)—supplemented with the cRAP contaminant database. Search parameters included trypsin specificity (full digestion), a maximum of two missed cleavage sites, a 10 ppm precursor mass tolerance, and a 0.02 Da fragment mass tolerance. Carbamidomethylation of cysteine (+57.021 Da) was set as a static modification, while oxidation of methionine (+15.995 Da) was considered a dynamic modification. Peptide-spectrum matches (PSMs) were validated using the Percolator node; only identifications with a strict false discovery rate (FDR) below 1% at both the peptide and protein levels were retained. Protein grouping followed the strict parsimony principle to report the minimum number of proteins necessary to explain all observed peptides. For label-free quantification (LFQ), LC-MS features were extracted from MS1 precursor scans using the Minora Feature Detector node. Protein abundances were calculated by summing the precursor ion intensities (peak area) of both unique and razor peptides identified via MS2 fragmentation spectra. Final abundances were normalized to the total peptide amount per sample and scaled to an average of 100 to account for loading variations.

#### LC-MS/MS analysis

Peptides were analyzed using a Vanquish Neo nano-HPLC system coupled to an Orbitrap Exploris 480 mass spectrometer (Thermo Fisher Scientific) operating in data-dependent acquisition (DDA) mode. A 1 µL injection of peptide solution was first trapped on a C18 column (Acclaim PepMap 100, 3  µm, 75  µm × 2  cm) at 5 µL/min. Separation was performed on an analytical C18 column (PepMap 100, 3 µm, 75 µm × 15  cm) at a flow rate of 300 nL/min using a gradient of 0.1% formic acid (solvent A) and 80% ACN with 0.08% formic acid (solvent B). Full MS spectra were acquired at a resolution of 60,000 (at 200 m/z) with an automatic gain control (AGC) target of 3 × 10^6^ ions. MS/MS fragmentation was performed using higher-energy collisional dissociation (HCD).

#### Data processing and functional annotation

Raw data were processed using Proteome Discoverer (v2.5.0.400). Protein identification was performed using the Sequest HT search engine against the proteomes of the four co-cultured species (*P. koreensis*, *N. cinerea*, *Rothia aeria*, and *S. downei*) and the cRAP contaminant database. Search parameters included the enzyme trypsin (full) with ≤2 missed cleavages. Tolerances of 10 ppm precursor and 0.02 Da fragment mass tolerance. Modifications included static carbamidomethylation of cysteine (+57.021 Da) and dynamic oxidation of methionine (+15.995 Da). Peptide-spectrum matches (PSMs) were validated using the Percolator node (FDR < 1%). For label-free quantification (LFQ), LC-MS features were extracted using the Minora Feature Detector node. Protein abundances were calculated by summing the precursor ion intensities of unique and razor peptides, normalized to total peptide amount, and scaled to an average of 100. To identify protein functions, sequences were uploaded to the Kyoto Encyclopedia of Genes and Genomes (KEGG) via GhostKoala (https://www.genome.jp/kegg/) for the assignment of KEGG Orthology (KO) terms. For downstream analysis, only biological pathways containing at least five proteins were considered for further evaluation. The relative abundance of each species was determined by calculating the summed intensity of all proteins associated with each microorganism. This value was expressed as a proportion relative to the total intensity of all bacterial proteins identified in each sample.

### Modified agar diffusion test

In order to investigate possible antagonistic effects of the bacterial strains against each other, we performed a modified agar diffusion test. For this test, 100 µL of each monoculture was spread onto a BHI agar plate. After drying, sterile paper filter discs were placed onto the agar plates and inoculated with 20 µL of the other strains. The plates were incubated overnight at 34°C, and possible inhibition zones were evaluated.

### pH trajectories

Mono- and co-cultures were incubated for 10 days at 34°C with constant shaking at 200 rpm. Each day, a 100 µL aliquot was collected into an Eppendorf tube for pH determination. The pH was measured using Chemsolute universal pH indicator paper (range 1–14).

### Acid perturbation

To assess the co-culture’s response to disturbance, overnight cultures were used to inoculate a 50 mL co-culture in BHI medium at an initial density of 1 × 10⁷ cells/mL per strain, followed by incubation at 34°C under continuous shaking at 200 rpm. pH was recorded daily, and viable counts (CFU/mL) were determined at selected time points. After inoculation (t0), the co-culture was maintained without treatment until day 2, when the pH had stabilized at 9–10. On day 2, sterile-filtered 0.3% (vol/vol) acetic acid was added to induce an abrupt pH decrease, and the pH was evaluated immediately after addition. Two hours after acid exposure, a 500 µL aliquot of the acid-treated co-culture was transferred into 50 mL fresh BHI medium as a control. pH readjustment was then monitored by daily pH measurements, with CFU/mL determined at the start and end of the experiment. Resistance (RS) was calculated as follows:


$$RS=1-\frac{2|y0-yL|}{y0+|y0-yL|}.$$

Resilience (RL) was calculated as follows:


$$RL=\left[\frac{2|y0-yL|}{|y0-yL|+|y0-yn|}-1\right]÷(tn-tL).$$

### Comparative genomics

To compare the metabolic potential of the four strains *P. koreensis*, *R. aeria*, *S. downei*, and *N. cinerea*, metabolic reconstruction was performed using the gapseq (v1.2) algorithm with default settings. As input files, type strain-specific genomes available from the National Center for Biotechnology Information (NCBI) were used. The pathway output files for each strain were filtered for present pathways and compared using Python, creating files for shared and unique pathways. These pathways were then grouped into superclasses according to the MetaCyc classification hierarchy.

### Bioinformatic investigation of siderophore receptors

The investigation of the putative siderophore receptors was conducted using the protein-protein blast from NCBI. Hand-curated siderophore receptors found in *P. koreensis* were compared to the annotated *N. cinerea* genome, and the 3D structure of the best hit was predicted using AlphaFold2. The predicted 3D structures were then compared to all of the 3D structures present in the Protein Data Bank (PDB) through a heuristic search using the DALI server. All input and output files can be found in Data S6.

### Statistical analysis

Statistical analyses were performed using GraphPad Prism (v9.5). For one-way and two-way ANOVA analyses, we set the level of significance * to *P* < 0.05. The Bray–Curtis dissimilarity index was computed and visualized as non-metric multidimensional scaling (NMDS) analysis with Python (v3.9), using the packages pandas, scikit-learn, Matplotlib, and NumPy. Pathway distribution was computed and visualized with Python 3.9 using the packages pandas, Matplotlib, and UpsetPlot. ChatGPT-4o was used to help generate Python scripts. The analysis scripts can be found in Data S7.

## Data Availability

Files S1–S8 are deposited in an online repository under https://doi.org/10.5281/zenodo.21510789.

## SUPPLEMENTAL MATERIAL

Supplemental Legends msystems.00491-26-s0001.docx Legends for Data S1, S3, and S4.

Data S1 msystems.00491-26-s0002.xlsx ANOVA results of obtained growth curves.

Data S2 msystems.00491-26-s0003.docx Growth curves of bacterial strains used for compositional resilience testing.

Data S3 msystems.00491-26-s0004.xlsx Metaproteome table of the co-culture cell pellet on day 10 of cultivation.

Data S4 msystems.00491-26-s0005.xlsx ANOVA of Bray-Curtis dissimilarity index of the consortium.

Data S5 msystems.00491-26-s0006.docx Pathway predictions made using gapseq of the community members.

Data S6 msystems.00491-26-s0007.docx Link to depository where the AlphaFold2 output files from the structural alignment of the siderophore receptors can be found.

Data S7 msystems.00491-26-s0008.docx Python codes used for data analysis.

Data S8 msystems.00491-26-s0009.docx Details of metaproteome analysis.

ASM does not own the copyrights to Supplemental Material that may be linked to, or accessed through, an article. The authors have granted ASM a non-exclusive, world-wide license to publish the Supplemental Material files. Please contact the corresponding author directly for reuse.
