# Supplementary material for: Establishment of an in vitro aerobic bacterial community as a model of the human lung microbiome
Source: mSystems. 2026 Aug 3;11(9):e00491-26. doi: 10.1128/msystems.00491-26 (PMC13595950; doi:10.1128/msystems.00491-26)
Supplement: Supplementary file 1 [file msystems.00491-26-s0001.docx]

**Supplemental Legend S1, S3, S4**

**Establishment of an *In Vitro* Aerobic Bacterial Community as a Model of the Human Lung Microbiome**

Maria Kulosa^1^, Matheus Regis Belisário-Ferrari^1^, Florian Semmler^1^, Mathilde Büttner^1^, Corinna Duck^1^, Zeinab Namazi^2^, Nico Jehmlich^2^, Martin von Bergen^2^, Tobias Bonitz^1^, Leonard Kaysser^1^

Legend S1:

Growth curve data of P. koreensis, R. aeria, S. downei, and N. cinerea in Brain-Heart Infusion (BHI) medium over 36 hours, derived from two biological replicates each with four technical replicates. The second sheet presents ANOVA results comparing monoculture growth of each strain to their growth in a four-species co-culture.

Legend S3:

Raw metaproteomic data from a four-species co-culture of P. koreensis, R. aeria, S. downei, and N. cinerea after 10 days of cultivation in Brain-Heart Infusion (BHI) medium.

Legend S4:

Bray–Curtis dissimilarity index (BCDI) data calculated from three qPCR measurements ofa co-culture of *P. koreensis*, *R. aeria*, *S. downei*, and *N. cinerea* over a 10-day co-cultivation time course (sheet 1). The data were used for repeated-measures one-way ANOVA, its results are found in sheets 2-5.
