# Supplementary material for: Establishment of an in vitro aerobic bacterial community as a model of the human lung microbiome
Source: mSystems. 2026 Aug 3;11(9):e00491-26. doi: 10.1128/msystems.00491-26 (PMC13595950; doi:10.1128/msystems.00491-26)
Supplement: Supplementary file 2 [file msystems.00491-26-s0003.docx]

**Supplementary Data S2: Growth curves**

Growth curves of *C. durum*, *S. turicensis*, *R. erythropolis* and *L.mirabilis* as well as growth curves of the original consortium supplemented with each new strain separately and as a fully extended co-culture.

(**A**) Growth curves determined by measuring OD with a spectrophotometer and calculating the actual OD by dilution over 50 h in 25 mL BHI medium. Values are from two technical replicates ± SEM. (**B**) Growth curves based on automated optical density measurements recorded in a plate reader over 73 h in 1 mL BHI medium. Values are from three technical replicates ± SEM. (**C**) Growth curves of the extended co-cultures over 36 hours in 1 mL BHI medium. Comparison of growth of the established co-culture, co-cultures extended by one additional strain each, and the co-culture extended with all four new strains (co-culture + 4). Values represent three technical replicates ± SEM.
