# Supplementary material for: Establishment of an in vitro aerobic bacterial community as a model of the human lung microbiome
Source: mSystems. 2026 Aug 3;11(9):e00491-26. doi: 10.1128/msystems.00491-26 (PMC13595950; doi:10.1128/msystems.00491-26)
Supplement: Supplementary file 4 [file msystems.00491-26-s0007.docx]

**Establishment of an *In Vitro* Aerobic Bacterial Community to Model Human Lung Microbiome Dynamics**

Maria Kulosa^1^, Matheus Regis Belisário-Ferrari^1^, Florian Semmler^1^, Mathilde Büttner^1^, Corinna Duck^1^, Zeinab Namazi^2^, Nico Jehmlich^2^, Martin von Bergen^2^, Tobias Bonitz^1^, Leonard Kaysser^1^

Correspondance:
maria.kulosa@medizin.uni-leipzig.de; leonard.kaysser@medizin.uni-leipzig.de

**Supplementary file S6 can be found under this link:** [https://doi.org/[10.5281/zenodo.15871295](https://doi.org/10.5281/zenodo.15871295)](https://doi.org/10.5281/zenodo.15871296)
