# Supplementary material for: Establishment of an in vitro aerobic bacterial community as a model of the human lung microbiome
Source: mSystems. 2026 Aug 3;11(9):e00491-26. doi: 10.1128/msystems.00491-26 (PMC13595950; doi:10.1128/msystems.00491-26)
Supplement: Supplementary file 5 [file msystems.00491-26-s0008.docx]

**Supplementary data 7: python scripts for data analysis**

**1. Upset Plot based on pathway prediction**

import pandas as pd

import upsetplot

def load_and_filter_data(filepath):

try:

df = pd.read_csv(filepath, sep=";", skiprows=3)

\# Explicitly convert 'Prediction' column to string to ensure .str methods work

df['Prediction'] = df['Prediction'].astype(str)

\# Now safely apply .str methods after conversion

filtered_df = df[df['Prediction'].str.strip().str.lower() == 'true']

return set(filtered_df['Name'].unique())

except FileNotFoundError:

print(f"File not found: {filepath}")

return set()

except KeyError as e:

print(f"Missing column in file {filepath}: {e}")

return set()

\# Paths to the data files for each bacteria

\# Load and filter data for each bacteria

rothia_pathways = load_and_filter_data(rothia_filepath)

pseudomonas_pathways = load_and_filter_data(pseudomonas_filepath)

streptococcus_pathways = load_and_filter_data(streptococcus_filepath)

neisseria_pathways = load_and_filter_data(neisseria_filepath)

\# Calculate unique and shared pathways among all combinations of bacteria

shared_all_four = rothia_pathways & pseudomonas_pathways & streptococcus_pathways & neisseria_pathways

\# Calculating unique pathways for each bacteria by subtracting pathways found in any other bacteria

unique_rothia = rothia_pathways - (pseudomonas_pathways | streptococcus_pathways | neisseria_pathways)

unique_pseudomonas = pseudomonas_pathways - (rothia_pathways | streptococcus_pathways | neisseria_pathways)

unique_streptococcus = streptococcus_pathways - (rothia_pathways | pseudomonas_pathways | neisseria_pathways)

unique_neisseria = neisseria_pathways - (rothia_pathways | pseudomonas_pathways | streptococcus_pathways)

\# Display counts for unique and shared pathways as an example of how to summarize the analysis

print(f"Shared among all four bacteria: {len(shared_all_four)}")

print(f"Unique pathways in Rothia: {len(unique_rothia)}")

print(f"Unique pathways in Pseudomonas: {len(unique_pseudomonas)}")

print(f"Unique pathways in Streptococcus: {len(unique_streptococcus)}")

print(f"Unique pathways in Neisseria: {len(unique_neisseria)}")

import pandas as pd

from upsetplot import UpSet, from_contents

\# Combine all unique pathways across all bacteria for indexing

all_pathways = rothia_pathways.union(pseudomonas_pathways, streptococcus_pathways, neisseria_pathways)

\# Convert set to list for DataFrame index

all_pathways_list = list(all_pathways)

\# Initialize a DataFrame with False values for presence/absence of pathways in each bacterium

upset_data = pd.DataFrame(index=all_pathways_list, columns=['Rothia', 'Pseudomonas', 'Streptococcus', 'Neisseria'], dtype=bool)

upset_data.fillna(False, inplace=True)

\# Populate the DataFrame with True if the pathway is present in the bacterium

for pathway in all_pathways:

upset_data.loc[pathway, 'Rothia'] = pathway in rothia_pathways

upset_data.loc[pathway, 'Pseudomonas'] = pathway in pseudomonas_pathways

upset_data.loc[pathway, 'Streptococcus'] = pathway in streptococcus_pathways

upset_data.loc[pathway, 'Neisseria'] = pathway in neisseria_pathways

\# Define the contents based on presence/absence in the upset_data DataFrame

contents = {

'R. aeria': set(upset_data[upset_data['Rothia']].index),

'P. koreensis': set(upset_data[upset_data['Pseudomonas']].index),

'S. downei': set(upset_data[upset_data['Streptococcus']].index),

'N. cinerea': set(upset_data[upset_data['Neisseria']].index),

}

\# Define the contents based on presence/absence in the upset_data DataFrame

contents = {

r'$\it{R. aeria}$': set(upset_data[upset_data['Rothia']].index),

r'$\it{P. koreensis}$': set(upset_data[upset_data['Pseudomonas']].index),

r'$\it{S. downei}$': set(upset_data[upset_data['Streptococcus']].index),

r'$\it{N. cinerea}$': set(upset_data[upset_data['Neisseria']].index),

}

import matplotlib.pyplot as plt

\# Use from_contents to correctly format the data for UpSet

with warnings.catch_warnings():

warnings.simplefilter("ignore", category=FutureWarning)

example = from_contents(contents)

upset = UpSet(example, subset_size='count', show_counts=True, sort_by='cardinality')

upset.plot()

upset = UpSet(example)

upset = UpSet(example, show_counts=True, sort_by='cardinality')

upset.style_subsets(min_degree=1, facecolor="blue")

upset.style_subsets(min_degree=2, facecolor="purple")

upset.style_subsets(min_degree=3, facecolor="red")

upset.style_subsets(min_degree=4, facecolor="green")

upset.plot()

plt.ylabel('Number of Pathways') # Change y-axis label

plt.show()

**2. Bray-Curtis-Dissimilarity-Index and Non-Metric Multidimensional Scaling Analysis**

import pandas as pd

from sklearn.metrics import pairwise_distances

from sklearn.manifold import MDS

import matplotlib.pyplot as plt

import numpy as np

from matplotlib.patches import Ellipse

\# Load the data

file_path = r'D:\NMDS\qPCR_NMDS (1).csv'

data = pd.read_csv(file_path, sep=';', decimal=',')

\# Separate days from strains and replicates

days = data['d']

strains_replicates = data.drop(columns=['d'])

\# Replace commas with dots and convert string numbers to floats

strains_replicates = strains_replicates.apply(lambda x: x.str.replace(',', '.').astype(float) if x.dtype == 'O' else x)

\# Calculate Bray-Curtis dissimilarity matrix

bray_curtis_distance = pairwise_distances(strains_replicates.T, metric='braycurtis')

print("Bray-Curtis Dissimilarity Matrix:\n", bray_curtis_distance)

\# Perform Non-metric Multidimensional Scaling (NMDS)

mds = MDS(n_components=2, dissimilarity='precomputed', random_state=42)

nmds_result = mds.fit_transform(bray_curtis_distance)

\# Get the stress of the NMDS calculation

stress = mds.stress_

\# Define strain labels

strain_labels = ['P. koreensis', 'R. aeria', 'S. downei', 'N. cinerea']

\# Create a color map for strains

colors = ['blue', 'red', 'green', 'orange']

color_map = {strain: colors[i] for i, strain in enumerate(strain_labels)}

\# Plot the NMDS result with ellipsoids

plt.figure(figsize=(10, 6))

\# Plot individual points and ellipsoids

for label in strain_labels:

indices = [i for i, col in enumerate(strains_replicates.columns) if col.startswith(label)]

indices = np.array(indices)

if len(indices) > 0:

plt.scatter(nmds_result[indices, 0], nmds_result[indices, 1], c=color_map[label], s=50, label=label)

\# Plot ellipsoid

mean_pos = np.mean(nmds_result[indices], axis=0)

covariance_matrix = np.cov(nmds_result[indices], rowvar=False, bias=True)

eigenvalues, eigenvectors = np.linalg.eig(covariance_matrix)

angle = np.degrees(np.arctan2(*eigenvectors[:, 0][::-1]))

ellipsoid = Ellipse(mean_pos, 2 * np.sqrt(eigenvalues[0]), 2 * np.sqrt(eigenvalues[1]), angle=angle, alpha=0.1,

color=color_map[label])

plt.gca().add_patch(ellipsoid)

plt.xlabel('NMDS1')

plt.ylabel('NMDS2')

plt.grid(False)

plt.legend()

plt.xlim(-0.15, 0.20)

plt.ylim(-0.20, 0.20)

plt.show()

print("Stress of the NMDS calculation:", stress)

3. Stacked bar graph metaproteome analysis

import matplotlib.pyplot as plt

from matplotlib.lines import Line2D

species = ['P. koreensis', 'N. cinerea', 'R. aeria', 'S. downei']

percent_abundance = [33.19, 19.67, 42.33, 4.80]

colors = {

'P. koreensis': '#4F81BD',

'N. cinerea': '#F79646',

'R. aeria': '#C0504D',

'S. downei': '#9BBB59'

}

fig, ax = plt.subplots(figsize=(7, 2)) # normale Breite, flache Höhe

bar_height = 0.07

y = ['Protein abundance']

left = 0

for sp, perc in zip(species, percent_abundance):

ax.barh(y, perc, bar_height, left=left,

color=colors[sp], edgecolor='black', linewidth=0.5)

ax.text(left + perc / 2, 0, f"{perc:.1f}", va='center', ha='center',

color='black', fontsize=9)

left += perc

ax.set_xlabel('Value [%]')

ax.set_title('Protein abundance per species')

ax.set_xlim(0, 100)

ax.set_yticks([])

custom_legend = [Line2D([0], [0], color=colors[sp], lw=10) for sp in species]

labels = [r"$\it{" + sp + "}$" for sp in species]

ax.legend(custom_legend, labels, title='Species', bbox_to_anchor=(1.05, 1), loc='upper left')

plt.tight_layout()

plt.show()
