# Supplementary material for: Establishment of an in vitro aerobic bacterial community as a model of the human lung microbiome
Source: mSystems. 2026 Aug 3;11(9):e00491-26. doi: 10.1128/msystems.00491-26 (PMC13595950; doi:10.1128/msystems.00491-26)
Supplement: Supplementary file 6 [file msystems.00491-26-s0009.docx]

**Supplementary Data S8: Metaproteome analysis**

**A**

**Table 1: Protein groups in mono- and co-culture samples.**

| Category | Proteins | Protein Groups | Peptide Groups | PSMs | MS/MS Spectrum |
| --- | --- | --- | --- | --- | --- |
| co | 4444 | 4323 | 27982 | 225650 | 221422 |
| mono | 5048 | 4929 | 48376 | 288141 | 380464 |

**B**

**Figure 1: Shared and exclusive proteins in mono- and co-culture.**

**C**

**Figure 2 Differential Protein Expression: Co-culture vs. Mono-culture Across Species.**
